# Characterization of the Physical Chemistry Properties of Iron-Tailing-Based Ceramsite

**DOI:** 10.3390/molecules28052258

**Published:** 2023-02-28

**Authors:** Shaoguang Hua, Dun Wu, Jian Wu, Shuqin Li, Guijian Liu, Dejian Pei

**Affiliations:** 1 School of Earth and Space Sciences, University of Science and Technology of China, Hefei 230026, China; 2 Sinosteel Maanshan General Institute of Mining Research Co., Ltd., Maanshan City 243000, China; 3 Key Laboratory of Intelligent Underground Exploration, College of Civil Engineering, Anhui Jianzhu University, Hefei 230601, China

**Keywords:** iron tailings ceramsite, internal structure, mechanical properties, adsorption

## Abstract

In order to deal with the problems of resource waste and environmental pollution caused by solid waste, iron tailings (mainly SiO_2_, Al_2_O_3_ and Fe_2_O_3_) were used as the main raw material to create a type of lightweight and high-strength ceramsite. Iron tailings, dolomite (industrial grade, purity 98%) and a small amount of clay were combined in a N_2_ atmosphere at 1150 °C. X-ray fluorescence spectrometry (XRF), X-ray diffraction (XRD), scanning electron microscopy (SEM), energy dispersive X-ray spectroscopy (EDS) and a themogravimetric analysis (TGA) were performed and the specific surface area was analyzed to determine the strength and adsorption of the ceramsite. The results of the XRF showed that SiO_2_, CaO and Al_2_O_3_ were the main components of the ceramsite, with MgO and Fe_2_O_3_ also included. The results of the XRD and SEM-EDS showed that the ceramsite contained several kinds of minerals and was mainly composed of akermanite, gehlenite and diopside, and that the morphology of the internal structure of the ceramsite was mainly massive and contained a small number of particles. The ceramsite could be used in engineering practice to improve the mechanical properties of materials and meet the requirements of actual engineering for the strength of materials. The results of the specific surface area analysis showed that the inner structure of the ceramsite was compact and that there were no large voids. The voids were mainly medium and large, with a high stability and strong adsorption ability. The TGA results showed that the quality of the ceramsite samples will continue to increase within a certain range. According to the XRD experimental results and experimental conditions, it was speculated that in the part of the ore phase containing Al, Mg or Ca in the ceramsite, the elements underwent relatively complex chemical reactions with each other, resulting in the formation of an ore phase with a higher molecular weight. This research provides the basis of characterization and analysis for the preparation of high-adsorption ceramsite from iron tailings and promotes the high-value utilization of iron tailings for waste pollution control.

## 1. Introduction

With the acceleration of urbanization in China, the development of the mining industry has driven an increase in ore consumption, leading to the accelerated growth rate of solid waste. With an increase in the emphasis on environmental protection, the secondary utilization of solid waste has gradually become a focus [1,2]. In the process of mineral resources development, a large number of tailings will be discharged. The increasing number of accumulated tailings will not only occupy large amounts of land, thus polluting the environment, but will also bring huge safety risks, endangering people’s lives and properties [3,4]. Since April 2005, China has implemented the Law of the People’s Republic of China on the Prevention and Control of Environmental Pollution by Solid Waste and established the principle of “reduction, recycling and harmless” in the prevention and control of solid waste pollution [5]. Therefore, in order to improve the effective disposal of solid waste and explore more reasonable methods of utilizing solid waste, the recycling of solid waste in an environmentally friendly and sustainable way has been imminent [6]. In 2006, according to national statistics, the annual output of iron ore tailings was approximately 1.99 billion tons. This number was increasing at the rate of more than 300 million tons per year [7,8]. These statistics include iron tailings discharged from mines, steel slag and copper slag in smelting slag and a large amount of industrial solid waste such as coal gangue and domestic sludge from other industries [9,10]. The long-term storage of a large amount of solid waste not only occupies a large amount of land area, increasing the cost of construction, maintenance and management, but also seriously harms the environment and ecology [11]. In order to maintain a clean and safe human living environment, reduce the damage of mineral resources to the natural ecology and realize the harmonious development of mining production and society, it is necessary to carry out technical research on the coordination of various solid wastes for the comprehensive utilization of various solid wastes [12].

At present, there are few methods of using iron tailings to make ceramsite. Additionally, the studies on the valence changes, crystalline dispersion and transparent materials in the preparation process of ceramsite iron are not yet mature, and the physical characteristics of the main raw material of ceramsite are significantly affected by various elements in the tailings [13,14]. Considering the above situation, iron tailings are used as the main raw material that is fired into ceramsite and applied to wastewater treatment to realize waste pollution control and to open up a new method for the efficient resource utilization of iron tailings. At the same time, it also improves the economic benefits of ceramsite used for wastewater treatment and reduces the dependence of ceramsite on natural resources and manufacturing costs [15,16]. This will become a new research hotspot for establishing a reliable technical system for the collaborative preparation of ceramsite, using iron tailings as solid waste through conducting research with a theoretical basis, the exploration of process technology and performing small- and medium-sized tests for product verification. This research can truly transform waste into treasure, achieve ecological harmony, have significant social and environmental benefits and can meet the current urgent demand for solid waste utilization [17,18].

In this paper, the process of preparing ceramsite with iron tailings as the main raw material is first introduced. The mechanical properties and adsorption properties of the prepared ceramsite are then characterized and analyzed so as to provide effective evidence for ceramsite as a heavy metal adsorption material or detergent. The analysis methods included X-ray fluorescence (XRF), X-ray diffraction (XRD), scanning electron microscope (SEM), energy dispersive X-ray spectroscopy (EDS), themogravimetric analysis (TGA) and specific surface area analysis (the BET method) with the aim to provide new ideas for the efficient utilization of iron tailings as resources and in the realization of waste treatment.

## 2. Results and Discussion

### 2.1. XRD Analysis and SEM-EDS Analysis

**Figure 1:**
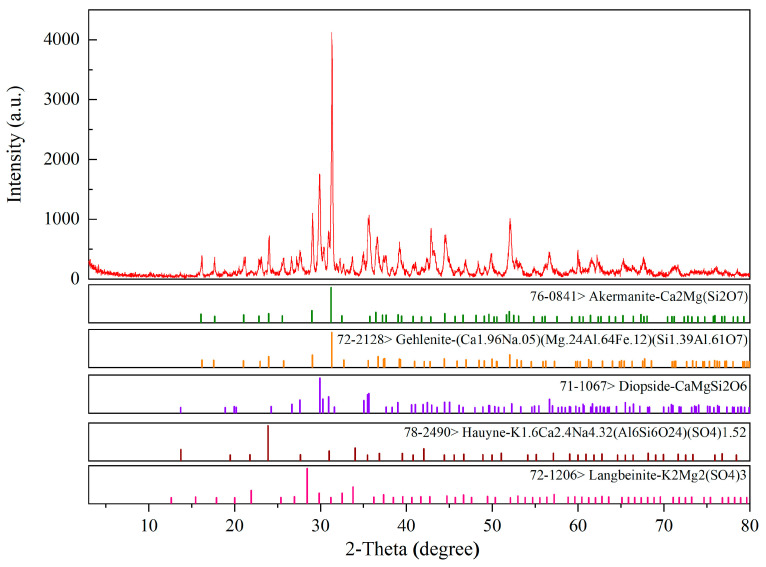
The XRD analysis results of the ceramsite.

To determine the phase composition of the baked ceramsite, an XRD analysis was performed. The results are shown in Figure 1. As can be seen from Figure 1, the number of diffraction peaks was large, and most of the peaks had low intensities. This may be because the main raw materials of ceramsite are iron tailings, dolomite and clay, and the mineral composition of the ceramsite prepared from these three raw materials was relatively complex. In the firing process, chemical reactions occurred between various raw materials, resulting in a large variety of minerals with a roughly equal content, resulting in low diffraction peaks and few peaks. It can be seen from the figure that the crystal phase in the ceramsite was mainly composed of three minerals: akermanite, gehlenite and diopside. In addition, there were two minor minerals: hauyne and langbeinite. According to the chemical molecular formula, the composition of the ceramsite minerals was consistent with the XRF results given in Table 1. Through a comparison of JCPDS cards, it was found that the diffraction peaks of the three minerals overlapped. It was speculated that the main causes were the raw materials and the firing temperature of the ceramsite. According to the experimental results of the XRF analysis in Section 2.3, there were many kinds of oxides in the raw materials. At the firing temperature of 1150 °C, various chemical reactions will occur between the raw materials, and the phase composition of ceramsite will change. This may lead to the overlapping of diffraction peaks. Pei et al. [19] prepared a new type of SiO_2_-Al_2_O_3_-CaO-MgO (10 wt%) ceramic with RM (red mud) as the main raw material, and their results also showed overlapping diffraction peaks. In addition, the research of Pei et al. showed that different sintering temperatures change the phase composition of the ceramics. It was speculated that changing the sintering temperature of the ceramsite in this paper would also lead to different diffraction peaks and that the phase composition would change.

Regarding the properties of the three phases, akermanite is colorless and is mainly composed of magnesium and calcium, with a high hardness and hydraulic properties [20]. Gehlenite has a high strength and good hydration resistance and can be used to improve the mechanical properties and hydration resistance of materials. It has a high calcium and silicon content in its components, and its color is mainly white or gray [21]. The diopside crystal structure is a monoclinic system, usually a columnar crystal. The aggregates are dense, massive, columnar, rod-shaped, granular and radial. It is grayish white in appearance and clean white after burning. It is an infertile material, and there are very small gaps inside [22]. Pei et al. [19] showed that the appearance of the above three minerals, especially diopside and anorthite, were the main crystal-phase components of the new ceramics. The ceramics with a higher RM (red mud) content (50 wt%) had the lowest optimal sintering temperature, the best physical properties and a flexural strength of 115.88 Mpa. Zhao et al. [23,24] used traditional ceramic raw materials (quartz, talc, clay and feldspar) as main raw materials. After adding steel slag, they successfully prepared new ceramics at the sintering temperature of 1200–1220 °C by using traditional ceramic technology. The crystal phases of the new ceramics were pyroxene group minerals including diopside, augite and diopside aluminian. The pyroxene group minerals in ceramic materials have excellent physical and mechanical properties, so the appearance of pyroxene group minerals in this study was conducive to enhancing the adsorption performance of ceramsite in engineering applications [25].

**Figure 2:**
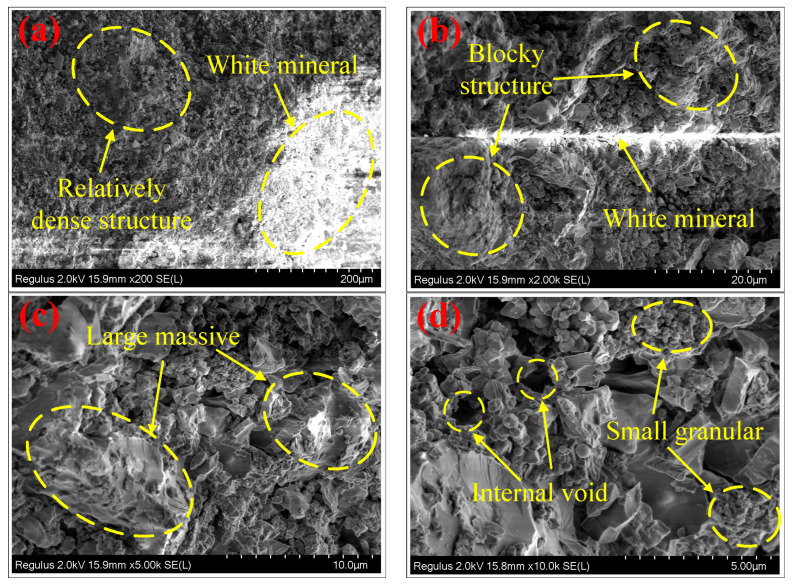
Results of the SEM analysis of the ceramsite. In the figure, In the figure, (**a**,**b**) represent the overall appearance of the interior of ceramsite under relatively small magnification, and (**c**,**d**) represent the local appearance of the interior of ceramsite under relatively large magnification.

After analyzing the phase composition of the ceramsite, the internal morphology was observed by SEM. The results are shown in Figure 2. As can be seen from Figure 2a, in the case of a small magnification, the color of the ceramsite grain was mainly dark gray and some parts were gray and white. Its structure was compact and dense with no obvious holes, providing a guarantee for the formation of a high-strength ceramsite. According to the experimental results of the XRD analysis and combined with the appearance characteristics of the three main minerals, akermanite, gehlenite and diopside, it was speculated that the white mineral in the figure was gehlenite or diopside. Figure 2b showed the results of Figure 2a after 10 times amplification. An observation of the figure showed that the internal structure of the ceramsite was mostly a massive structure with large and small blocks, demonstrating that the internal structure of the ceramsite was relatively homogeneous. In the middle of the figure a white mineral similar to the figure in Figure 2a also appeared. Further enlarging the microscope multiple obtained Figure 2c,d. Through the observation of Figure 2c, we noted the appearance of gehlenite or diopside more clearly. It was found that the surface of the block mineral was different, in a concave and convex shape, and the color was changed from the original gray and black to gray and white. In addition, granular minerals appeared in Figure 2c whose detailed structure could be observed from Figure 2d. According to the source of the raw materials, the cause of this granular structure may be the chemical reaction of the iron tailings and dolomite during calcination at a high temperature, producing Si particles and its compounds. This was also conducive to further improving the strength of the ceramsite structure. In Figure 2d, it can also be observed that there is a large gap inside the ceramsite with a diameter of approximately 5–10 μm. This provided a prerequisite for the good adsorption of the ceramsite.

**Figure 3:**
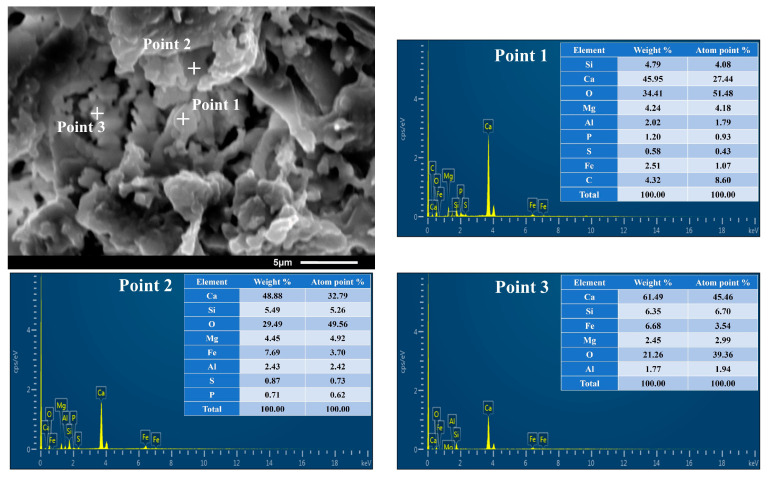
SEM-EDS analysis of internal morphology of the ceramsite.

In order to support the experimental results of the XRF and XRD, the SEM image with a magnification of 4000 was selected to obtain the SEM-EDS analysis diagram of the internal morphology of the ceramsite, shown in Figure 3. It can be seen from the figure that the elemental composition of the ceramsite was consistent with the chemical composition given by XRF, and the result was consistent with the three main minerals obtained by XRD analysis: akermanite, gehlenite and diopside. Regarding the newly emerging elements, P and C, it was speculated that the element C may be introduced by spraying carbon powder to enhance the conductivity during the SEM experiment, and the element P may come from the remaining 1.7% of oxides in XRF (Table 1). This was also consistent with the low content of element P in the EDS results.

### 2.2. Specific Surface Area Analysis

**Figure 4:**
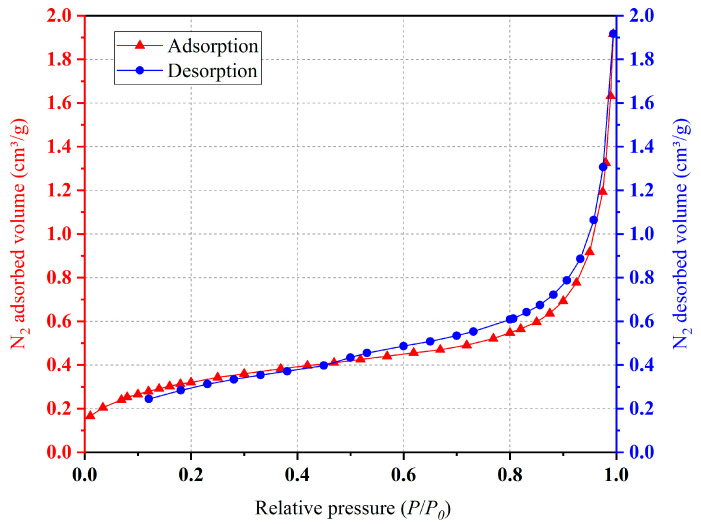
N_2_ adsorption/desorption isotherms of ceramsite.

**Figure 5:**
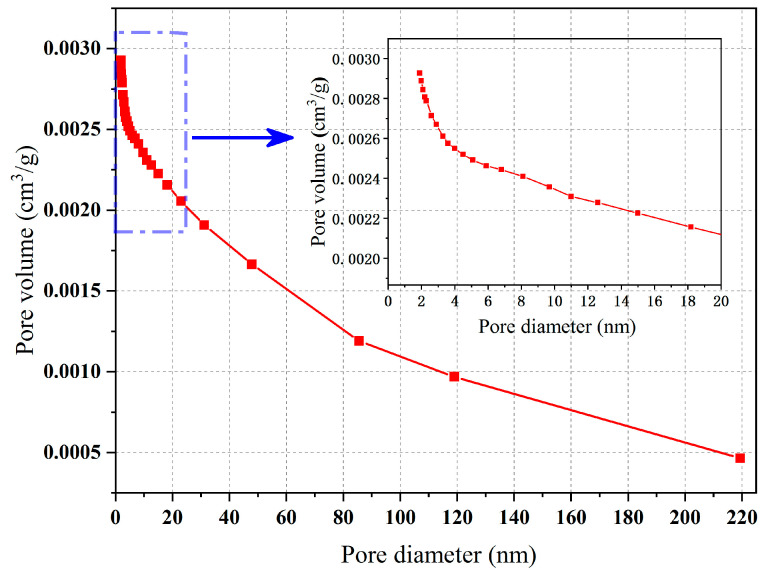
Pore size distribution of ceramsite.

After XRD analysis and the SEM-EDS analysis of ceramsite, the adsorption of the ceramsite was analyzed. According to the experimental method in Section 3.7, the adsorption equilibrium isotherm is shown in Figure 4. It can be seen from Figure 4 that the nitrogen adsorption isotherm of ceramsite prepared in this study increased slowly when the relative pressure was less than 0.6, and the growth rate began to accelerate when the relative pressure was greater than 0.6, increasing sharply when the relative pressure was greater than 0.9. According to the experimental results of the SEM, the reason for this phenomenon may be that when the relative pressure was 0 < P/P_0_ < 0.6, the number of micro pores in the ceramsite was lower and the contribution to the adsorption of N_2_ was less; therefore, the growth rate was slow. With the increase in the relative pressure (0.6 < P/P_0_ < 0.9), the increase in the number of medium-sized pores made the adsorption amount of N_2_ increase and the growth rate began to accelerate. When the relative pressure P/P_0_ > 0.9, the number of large pores made the adsorption rate of N_2_ increase sharply. The pore size distribution of the ceramsite was calculated according to the Barrett, Joyner and Halenda (BJH) models, as shown in Figure 5 [26]. This demonstrates that the pore size distribution segments of the ceramsite samples were relatively uniform. Most of them were between 0 and 220 nm. Among them, the medium and large pores with a pore size greater than 20 nm occupied the majority of the pore size distribution, which also provided a good explanation for the adsorption/desorption curve of N_2_ by the ceramsite. After calculation, the total pore volume reached 0.059 cm^3^/g.

The specific surface area was calculated according to the Brunauer–Emmett–Teller (BET) equation. The adsorption isothermal equation for BET is:

(1) $$\frac{1}{V\left(p_{0}/p-1\right)}=\frac{1}{V_{m}C}+\frac{C-1}{V_{m}C}\cdot \frac{P}{p_{0}}$$

where $p_{0}$ is the saturated vapor pressure of the adsorbate at the adsorption temperature, $V_{m}$ is the saturated adsorption capacity of the monolayer, and C is a constant. It can be seen from Formula (1) that when the experimental data is plotted according to $1/V\left(p_{0}/p-1\right)$ and $p/p_{0}$, a straight line can be obtained. The slope, *k*, of the straight line and the intercept, *b*, on the longitudinal axis are:

(2) $$k=\frac{C-1}{V_{m}C}$$

(3) $$b=\frac{1}{V_{m}C}$$

**Figure 6:**
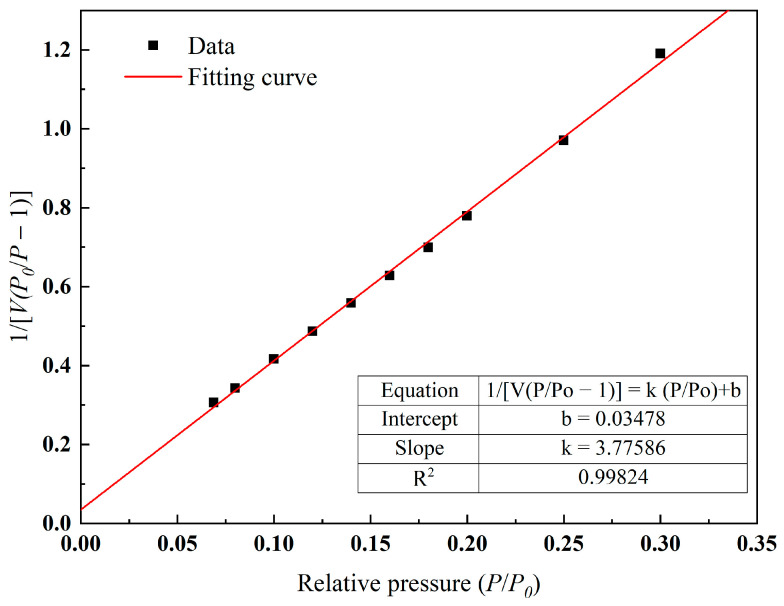
Calculation and analysis graph of specific surface area.

As shown in Figure 6, the line diagram obtained from the number of experiments in this paper showed that the slope and intercept of the line were 3.77586 and 0.03478, respectively. Simultaneous to Equations (2) and (3), substitute the values of *k* and *b* to obtain the values of $V_{m}$ and C, and then substitute them into the following equation:

(4) $$S=\frac{A_{m}N_{A}V_{m}}{22400}$$

where $A_{m}$ is the cross-sectional area of protons adsorbed in the standard state ($0.162\times 10^{-18}\;m^{2}$ in this paper) and $N_{A}$ is Avogadro’s constant ($N_{A}=6.02\times 10^{23}$ mol ^−1^). Substituting the values of *V_m_* and C into Formula (4), the specific surface area of ceramsite was calculated to be 1.1422 m^2^/g.

### 2.3. Themogravimetric Analysis

**Figure 7:**
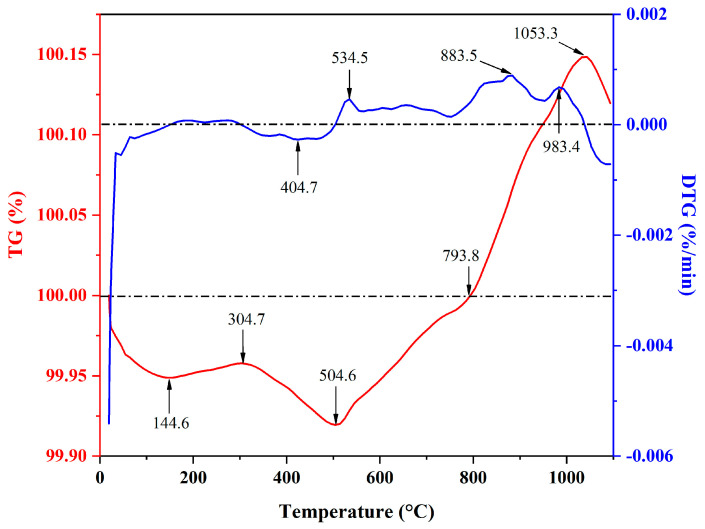
TG-DTG graph of ceramsite.

In the process of heating, inorganic substances will have chemical reactions at different temperatures. These reactions are closely related to the expansion of the ceramsite and the formation of the pore structure. After the ceramsite was prepared from iron tailings, dolomite and clay and was analyzed by thermogravimetry, the heat treatment characteristics of raw materials were studied by thermal conductivity gravimetric analysis. Specifically, TG represents the change of the residual mass of the matter with temperature, and DTG represents the weight loss rate corresponding to the TG curve. The ceramsite was crushed into a powder and subjected to thermogravimetric analysis experiments in N_2_ (100 mL/min) in the atmosphere and a heating rate of 10 °C/min to obtain the experimental results shown in Figure 7. As can be seen from Figure 7, the rate of mass loss within 50 °C was at a maximum and slowly decreased. At 100 °C, it began to stabilize. This may have been caused by the loss of free water and the physically adsorbed water in the pottery grain with the increase in temperature. When the temperature was 404.7 °C, the mass loss rate was relatively high, possibly due to the volatilization of bound water in the pellets, the burning and volatilization of organic matter or the decomposition of some existing salts. In addition, as can be seen from the figure, the quality of the ceramsite showed an overall decreasing trend before 500 °C, with only a small increase between 144.6 °C and 304.7 °C. However, after the temperature increased, an abnormal phenomenon can be observed from the TG curve. The mass of the ceramsite kept increasing after 504.6 °C, and it did not stop increasing until 1053.3 °C, when it began to decrease. Beginning from 793.8 °C, the total quality of the sample exceeded the original quality of the ceramsite sample. An analysis of the DTG curve showed that when the mass of the ceramsite increased continuously, the growth rate changed unevenly, reaching a maximum at 534.5 °C, 883.5 °C and 983.4 °C, respectively.

Combined with the results of similar previous studies [27,28,29,30,31], it was found that in most cases, although the sample mass will increase and decrease alternately at a certain temperature, the overall mass generally decreases. However, when the ceramsite samples presented in this article were tested at temperatures greater than 504.6 °C, the mass continued to increase. Finally, at 1053.3 °C, the mass ceased increasing and started to decrease (Figure 7). For this phenomenon, we speculated that, in part of the ore phase containing Al, Mg or Ca in the ceramsite, the elements underwent relatively complex chemical reactions with each other under the conditions of high temperature and N_2_, resulting in the formation of an ore phase with a higher molecular weight, leading to the weight increase of the sample. When the temperature of the thermogravimetric analysis reached 1053.3 °C, the chemical reaction of weight gain stopped, some raw materials decomposed at the subsequent temperature, and the sample quality began to decrease. This was also consistent with the fact that the generated iron oxide decomposed again.

## 3. Materials and Methods

### 3.1. Materials

The raw material in this paper, ceramsite, was mainly composed of industrial waste iron tailings, dolomite (calculated after loss on ignition) and clay (the purpose of which was to provide chemical elements for the equal formation of pyroxene in the target phase, improve the plasticity of the ceramsite green body and improve the strength of the ceramsite). Iron tailings was obtained from Anhui Magang Mining Resources Group Co., ltd (Maanshan, China), and dolomite and clay were taken from the surrounding area. In order to understand their chemical composition, the composition of the three raw materials was determined by X-ray fluorescence (XRF). The results are shown in Table 2. It can be seen that the iron tailings and clay were mainly composed of SiO_2_ and Al_2_O_3_, and the content of Fe_2_O_3_ in the iron tailings was also high. The dolomite was mainly composed of CaO and MgO and the content of other metal oxides was low, indicating that its purity was high. In addition, according to the table, the alkali metal oxide content of the three raw materials was low. The phase compositions of the three raw materials were as follows: (1) the iron tailings mainly contained albite phase, calcian phase, quartz phase, clinochlore phase and pyrite phase; (2) the dolomite was of industrial grade. The phase analysis showed that it was mainly dolomite phase with a high purity; and (3) the phase analysis showed that the clay phase was mainly quartz and kaolinite.

### 3.2. Ceramsite Preparation Process

**Figure 8:**
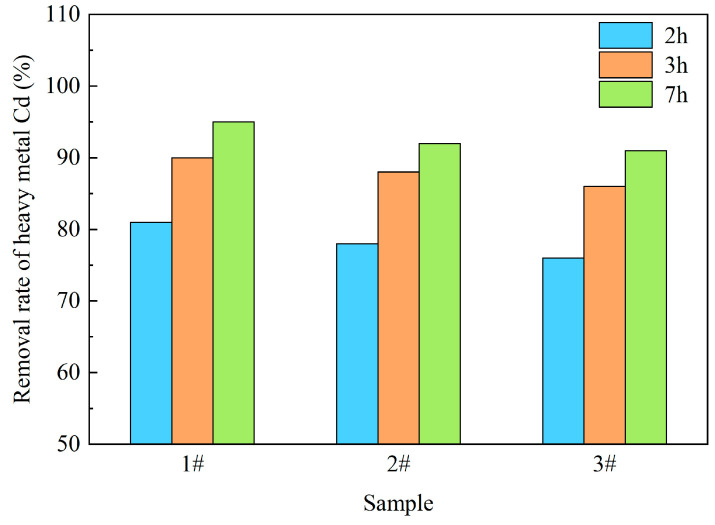
Cd removal rate of different samples under the same conditions.

In the actual experimental process, we prepared three kinds of Si-Ca-based ceramsite with different proportions under the same conditions. The ceramsite was prepared using iron tailings, dolomite and clay as raw materials through the phase diagram of SiO_2_-Al_2_O_3_-CaO-MgO [32]. The composition was based on the composition design shown in Table 3 (the preparation temperature was set at 1150 °C). Figure 8 shows the analysis and a comparison of the removal rate of heavy metal Cd by the three formulations under the same conditions. It can be seen from Figure 8 that with the passage of time, the removal rate of the three formulations of ceramsite for heavy metal Cd increased. Compared with the three formulations at the same time, it was found that the sample (Sample #1) characterized in this paper had the best purification effect, and that the removal rate of heavy metal Cd could reach 95%. Based on the above results, the purpose of this paper is to explain the excellent decontamination effect of this formulation of ceramsite through a number of targeted characterization analyses. Under the design of a Si-Ca-based ceramsite, the difference in the proportion of several raw materials will cause differences in the chemical composition of the green body. In the process of high-temperature phase reorganization, the phases were mainly pyroxene, cordierite and feldspar, with only a slight difference in the amount of formation. The generation process was similar to the report from the relevant literature [23,24,25]. Next, the preparation process of the ceramsite is introduced in detail.

The ceramsite raw material was mainly composed of industrial waste residue iron tailings, which accounted for 50% of the composition and were supplemented by 30% dolomite (according to the burn loss) and 20% clay. To improve the effect of ceramsite preparation, an additional 5% toner plays a reducing effect. The production and firing process of ceramsite were as follows: the raw material mixture of ceramsite and water were placed into a ball mill at a ratio of 1:1 for 20 min. They were then filtered through an 80-mesh sieve to obtain the slurry. The slurry was dried at 110 °C for 8 h, crushed to obtain powder, and an appropriate amount of water was added for granulation. The resulting material ball was dried at 110 °C for 2 h and fired in a tubular furnace. The firing atmosphere was nitrogen (250 mL/min), and the firing temperature rose from room temperature to 50 °C (5 °C/min), remained for 5 min to stabilize the front heating speed, rose from 50 °C to 1150 °C (10 °C/min), and, after 30 min at 1150 °C, decreased from 1150 °C to 500 °C (10 °C/min). It finally cooled to room temperature with the furnace.

**Figure 9:**
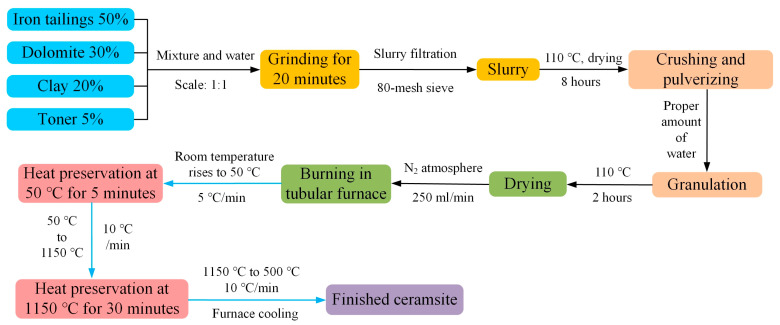
Preparation process of ceramsite.

Following the above steps one by one, the ceramsite density was 1.0 g/cm^3^. It had single diameter between 1.0 and 1.2 cm and a weight between 4.0 and 5.0 g. The ceramsite prepared according to the above steps had a flat ellipsoid shape, a gray-black color, a smooth surface and a uniform size. The preparation process for the ceramsite in this study is shown in Figure 9.

### 3.3. The XRF Method

According to the ceramsite preparation process in Figure 8, the finished iron tailing ceramsite was prepared at 1150 °C. In order to understand its chemical composition, an X-ray fluorescence spectrometer (XRF) was used to test the percentage content (%) of oxide in the formed ceramsite. The model of this instrument was XRF-1800 (manufacturer: SHIMADZU, Kyoto, Japan), the X-ray tube target was a rhodium target (Rh), the X-ray tube voltage was 60 KV, the X-ray tube current was 140 mA, and the X-ray tube power was 4 KVA. Before the experiment, the weight of the ceramsite powder sample was 3.0 g, and it passed through a 300-mesh sieve. The experimental results are shown in Table 1. According to Table 1, the constituent oxides of ceramsite were mainly SiO_2_, CaO and Al_2_O_3_. The rest were MgO and Fe_2_O_3_. After making the ceramsite, there was a certain oxide loss. After obtaining the oxide composition of the ceramsite, XRD, SEM-EDS, TGA were performed and the specific surface area was analyzed successively to investigate the strength and adsorption of the ceramsite. The results and discussion are shown in the following section.

### 3.4. The XRD Method

The mineral composition of ceramsite was tested using an X-ray diffractometer (instrument model: X ‘Pert MPD; manufacturer: PANalytical B.V., Almelo, The Netherlands). The X-ray light source was a Cu target, the instrument had a ceramic X-ray tube, the current voltage was 40 kV, the current was 40 mA, the goniometer was vertical goniometer, the goniometer radius was 240 mm, the minimum step length was 0.001°, each step took 0.2 s and the detector was a proportional detector. After the ceramsite was sufficiently ground before the experiment, the particle size of the powder was 200-mesh. The weight of the upper machine test sample was 2 g.

### 3.5. The SEM-EDS Method

Before observation with a scanning electron microscope, the ceramsite powder sample was stuck with conductive tape and a small medicine spoon of the sample was scattered on the conductive tape. The sample table was then lightly knocked off. The excess sample was finally blown with an ear ball. In this study, the tungsten filament scanning electron microscope (instrument model: EVO 18; manufacturer: Carl Zeiss, Jena, Germany) was used to observe the surface morphology of the powdered ceramsite.

### 3.6. The TGA Method

The thermal properties of the ceramsite were analyzed using a Thermogravimeter (instrument model: SDT Q600 V20.9 Build 20; manufacturer: TA Instruments, New Castle, DE, USA). The initial weight of the ceramsite sample was 7.96500 mg an it had a thermal rate of 10 °C/min. A programmed warming range from 19 °C to 1100 °C with a Nitrogen atmosphere was used.

### 3.7. The BET Method

The specific surface area was analyzed with a mesopore distribution analyzer (instrument model: Micromeritics Tristar II 3020; manufacturer: Micromeritics, Norcross, GA, USA) to represent the suction surface and absorption line, specific surface area (including the BET specific surface area, Langmuir specific surface area, etc.), pore size distribution (BJH, DFT model, etc.), pore volume and other information. During the isothermal adsorption test, the temperature of the analysis tank was −195.8 °C and the chemically inert gas N_2_ was used as the adsorbate. The sample mass was 0.5454 g and the gas adsorption capacity under different specific pressures, P/P_0_ (relative pressure, P is the real pressure of the gas, and P_0_ is the saturated vapor pressure of the gas at the measured temperature), was measured.

## 4. Conclusions

In this paper, a new type of ceramsite was prepared at 1150 °C in a N_2_ atmosphere using iron tailings as the main component, dolomite and a small amount of clay. Through a series of experiments such as XRF, XRD, SEM-EDS, TGA and a specific surface area analysis, the strength and adsorbability of the ceramsite were comprehensively investigated. The following conclusions were obtained:

(1) By analyzing the experimental results of the XRD, the ceramsite prepared in this paper contained gehlenite and diopside minerals with a high strength, demonstrating that this ceramsite had a higher strength and better hydration resistance than traditional ceramsite. It could be added to traditional building materials to achieve the dual effect of reducing the structural quality and enhancing the material strength;
(2) SEM-EDS analysis results showed that this type of ceramsite had a relatively dense structure inside, and that the internal structure was dominated by a massive structure. In addition, it contained a small amount of granular, simple minerals and voids. This provided evidence for the high strength of ceramsite and also reflected that the ceramsite had a good adsorbability;
(3) The experimental results of the specific surface area analysis showed that the pore size distribution of the internal pores of this type of ceramsite was relatively uniform, containing mainly medium and large pores. When adsorbing N_2_, the ceramsite showed “slow at first and then fast” characteristics, and its adsorbability was strong. Therefore, it could be considered for applications in the treatment of polluted wastewater and could play a role in specific environments;
(4) Thermogravimetric analysis showed that the quality of the ceramsite would continue to increase after being heated to 504.6 °C. According to the XRD experimental results and experimental conditions, it was speculated that, in part of the ceramsite ore phase containing Al, Mg or Ca, the elements underwent relatively complex chemical reactions with each other, resulting in the formation of an ore phase with a higher molecular weight.

## Figures and Tables

**Table 1:** Chemical composition of ceramsite samples (excluding toner).

Oxide Types	SiO_2_	CaO	Al_2_O_3_	MgO	Fe_2_O_3_	SO_3_	Na_2_O	K_2_O	Total
Percentage (%)	41.74	20.59	13.49	8.18	8.52	1.98	2.13	1.67	98.30

**Table 2:** Chemical composition of ceramsite raw materials (wt.%).

Raw Material	SiO_2_	CaO	Al_2_O_3_	MgO	Fe_2_O_3_	SO_3_	Na_2_O	K_2_O
Iron tailings	43.01	8.86	16.73	3.89	15.25	3.90	4.00	1.43
Dolomite	1.97	70.26	0.35	26.98	0.26	0.04	0.02	0.01
Clay	66.13	7.02	16.87	2.81	2.82	0.08	0.41	3.16

Note: 41.14% ignition loss of dolomite was not included.

**Table 3:** Raw material preparation scheme of Si-Ca-based ceramsite.

Sample	Iron Tailings	Dolomite	Clay
#1	50%	30%	20%
#2	60%	25%	15%
#3	70%	20%	10%

## Data Availability

Not applicable.

## Sample Availability

The data in the study can be obtained by applying to the author.
